# Evaluation of Movement Restriction of Spinal Orthoses Using Inertial Measurement Units

**DOI:** 10.3390/ijerph192416515

**Published:** 2022-12-08

**Authors:** Justyna Fercho, Michał Krakowiak, Rami Yuser, Tomasz Szmuda, Piotr Zieliński, Dariusz Szarek, Samuel D. Pettersson, Grzegorz Miękisiak

**Affiliations:** 1 Neurosurgery Department, Medical University of Gdansk, 80-214 Gdańsk, Poland; 2 Scientific Circle of Neurology and Neurosurgery, Neurosurgery Department, Medical University of Gdansk, 80-214 Gdańsk, Poland; 3 Department of Neurosurgery, Marciniak’s Hospital, 54-049 Wrocław, Poland; 4 Institute of Medicine, Opole University, 45-052 Opole, Poland

**Keywords:** spine, motion analysis, wearable sensors, biomechanics, brace, orthosis

## Abstract

Despite the frequent use of orthopedic braces or spine stabilizers in diseases such as kyphosis, lordosis, and scoliosis, as well as in the case of injuries and rehabilitation after surgeries, there is no clear evidence of their proper stabilization of the spine while carrying out daily activities. This study sought to assess the spine’s mobility while wearing three different orthopedic braces while performing basic tasks. Ten healthy subjects were enrolled. Three Inertial Measurement Units (IMUs) were attached superficially along the spine at approximate levels: cervical (C7), between thoracic (T8) and lumbar (L3), and sacrum. The angle between sensors was monitored to provide data on the sagittal profile. In addition, the displacement of the spine’s longitudinal axis was measured (rotation). There are three types of orthopedic braces: the semi-rigid Hohmann corset, the Jewett brace, and the Thoracolumbar Fixed Spinal Orthosis (TLSO). Four tasks were monitored: standing, sitting, walking, and picking up an item from the floor with one hand. All braces provided a similar level of stability in both the sagittal plane and rotational axis while lifting an object. On the other hand, while walking and sitting, the TLSO was the only orthosis providing a statistically significant rigidity in the sagittal plane. When performing a more voluntary task, the measured rigidity of softer braces was significantly increased when compared with more involuntary tasks. A certain degree of motion restriction with spinal orthoses may come from the feedback pressure, which stimulates paraspinal muscles to contract and thus increases the overall rigidity of the trunk.

## 1. Introduction

Spinal orthoses are external braces used as a primary or supplementary aid for vertebrae immobilization in various spinal disorders. Based on their function, orthoses can be divided into three categories: supportive orthoses (for temporary pain), immobilization orthoses (for severe pain, e.g., spinal trauma and post-op treatment), and corrective orthoses (primarily for pediatric patients with idiopathic spinal deformity). There are specific braces for each spine segment (e.g., cervical, thoracic, lumbar, sacral, and in combinations). Common medical conditions treated with bracing are fractures, spinal deformities, and non-specific low back pain. They are often used as a supplement after the surgical treatment of the thoracolumbar spine. In pediatric patients, orthotic therapy is often used to rectify congenital spinal deformity (idiopathic or related to neuromuscular diseases). Spinal bracing has three prime objectives [1]: pain control by restricting motion as well as by unloading specific structures of the spine (discs, vertebral bodies, facets); stabilizing weakened or damaged structures; and providing three-point support to correct and prevent progression of the deformity.

The effectiveness of bracing is highly controversial for most of the applications, except for low-grade adolescent deformity [2]. Although there is insufficient evidence to support the use of spinal orthoses in thoracolumbar burst fractures [3] and acute osteoporotic vertebral compression fractures [4], they are still in use due to the common-sense approach of immobilizing the injured organon. Our study was designed to evaluate the spine’s mobility while wearing three different types of orthopedic braces while performing certain rudimentary tasks.

## 2. Materials and Methods

This study includes 10 subjects from the Medical University of Gdansk. Inclusion criteria comprised no past medical history of spine diseases, spine surgery or injury; no thoracolumbar pain; no neurological deficits; and any other symptom that could indicate a pathological process compromising the spine’s mobility. Participants were in the healthy weight range (Body Mass Index (BMI) between 18.5 and 24.9) and were not burdened with diseases that could affect the musculoskeletal system or impair their efficiency. Each participant signed a written consent before contributing to this study. The local ethics committee approved the study at the Medical University of Gdansk (NKBBN/145/2021). The 9-axis MetaMotionR (Mbientlab, San Francisco, CA, USA) Inertial Measurement Units (IMUs) were firmly attached to three distinct points on the subject’s body. Each IMU is consisted of a 6-axis accelerometer + gyroscope and a 3-axis magnetometer for a real-time 9-axis sensor fusion. The first was attached to the most prominent spinous process in the cervical region (typically the C7 level), the second to the nadir of the lumbar concavity (typically at the L3 vertebra), and the third was attached midway between the two aforementioned points (around T8). Two values were then calculated: the difference between the C7 and L3 inclinations (C7L3) and between the T8 and L3 inclinations (T8L3), which translated into the inclinations of almost the entire TL spine and TL junction, respectively.

**Figure 1:**
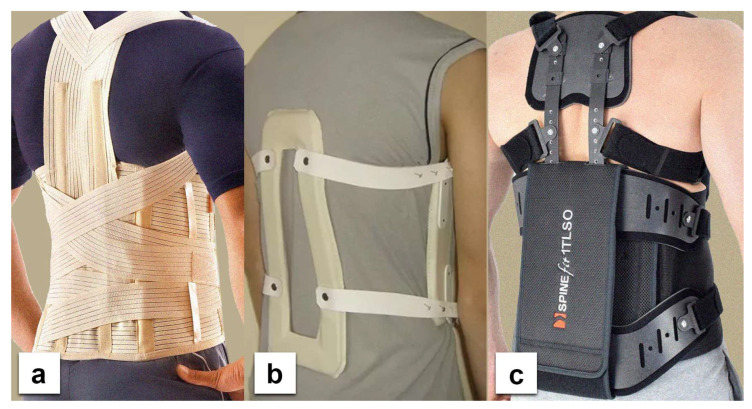
The orthoses used in the study (**a**)—Hohmann-type lace brace, (**b**)—Jewett orthosis, (**c**)—TLSO.

**Figure 2:**
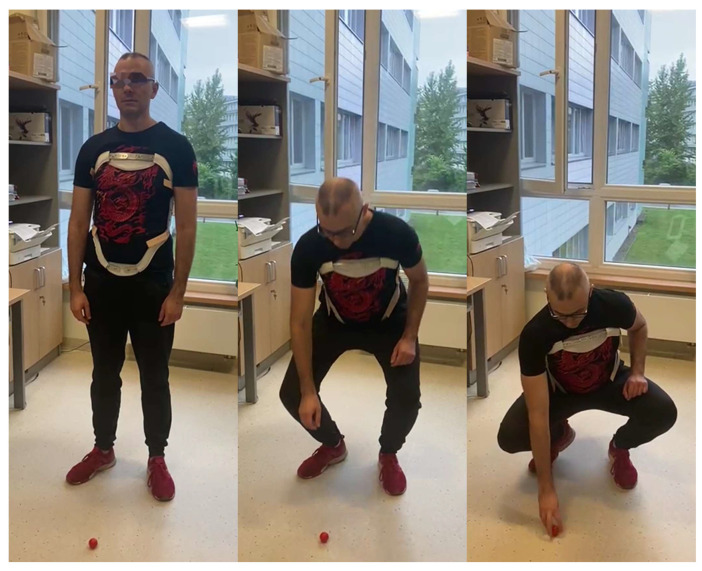
The task of lifting an object from the floor while wearing the Jewett brace.

The braces used in this study were: the Jewett brace, the Hohmann-type lace brace, and the Thoracolumbar Fixed Spinal Brace (TLSO) (Figure 1). The first one is a flexion control (hyperextension) three-point orthosis, the second is a semi-rigid corset, and the last is a tri-planar control device with a rigid frame. Each device was worn according to preset instructions and adjusted individually for each subject. The Physics Toolbox Sensor Suite Pro version 1.9.1 (MbientLab) was installed on the researcher’s smartphone and connected with the IMUs through Bluetooth to calibrate and register the measurements. The sampling frequency rate in the application was set to 100 Hz. Subjects were asked to perform the following tasks: stand, walk, sit, and lift an object from the ground using only one hand while wearing the above-mentioned braces (Figure 2). The data on sagittal displacement were analyzed with each task, and in addition, the relative rotation between sensors was measured for the lifting task. The baseline for the analysis of flexibility was the standing posture. Participants were also asked to fill out the five-item Likert-type questionnaire on subjective discomfort experienced while using each of the orthoses. The questionnaire that was used in this study is a significantly condensed version of one proposed by Curfts et al. [5] (Table 1).

## 3. Results

**Figure 3:**
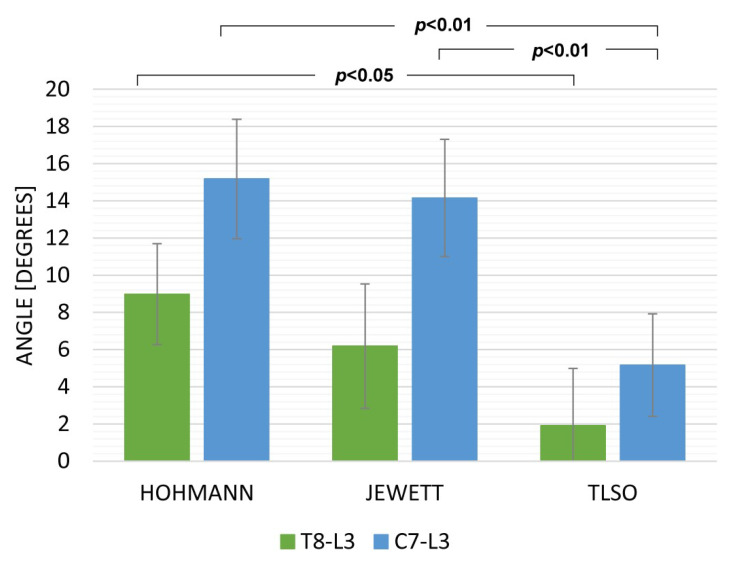
The maximum sagittal inclination during sitting.

**Figure 4:**
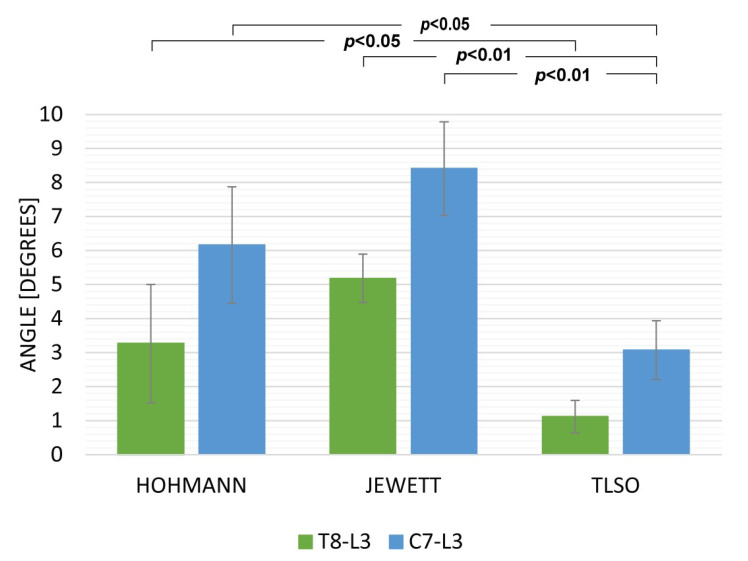
The maximum sagittal inclination while walking.

**Figure 5:**
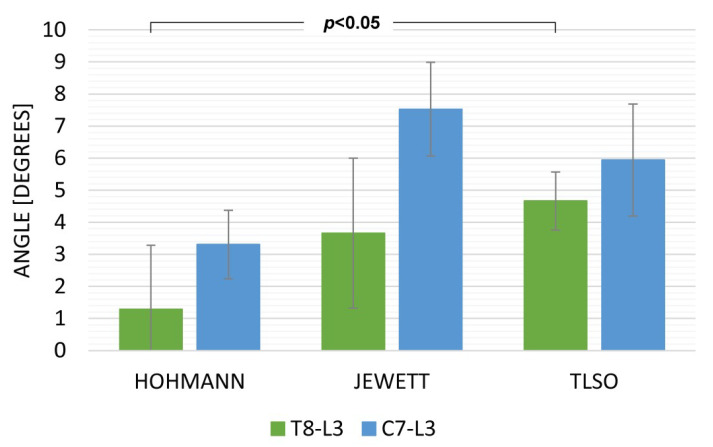
The maximum sagittal inclination while lifting an object from the floor.

**Figure 6:**
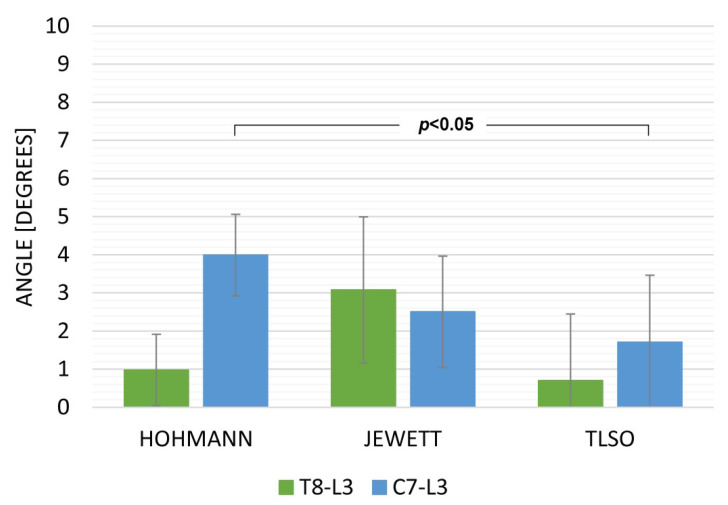
A graphical representation of the maximum axial rotation while lifting an object from the floor.

Data from the sitting and walking exercises revealed that the TLSO provided the highest limitation for both T8L3 and C7L3. The Hohmann brace was maximally displaced while sitting (Figure 3) with movement in C7L3 being 15.18 deg (SD 3.21) and in T8L3 being 8.97 deg. (SD 2.72). While walking, the TLSO provided the greatest restriction of movement in the sagittal plane (Figure 4). Mobility was minimal while using this orthosis: C7L3 was 3.07 deg. (SD 0.86), and T8L3 was 1.12 deg. (SD 0.47). The difference in the values for TLSO was statistically significant at *p* < 0.05. It was significantly different from the Hohmann brace (*p* < 0.05) and the Jewett brace (*p* < 0.01). Interestingly, for the dynamic task of lifting an object from the floor, the most rigid orthosis was the Hohmann brace, and TLSO was the least effective (Figure 5). The maximum displacement for the former at C7L3 was 1.29 deg. (SD 0.66) and at T8L3 was 3.31 deg. (SD 0.53). The difference between these two orthoses was statistically significant at *p* < 0.05. Referring to rotation, the movement in the TL area was nearly eliminated while utilizing both the Hohmann brace as well as the TLSO with T8L3 at 0.98 deg. (SD 0.93) and 0.69 deg. (SD 0.31), respectively (Figure 6). However, the difference was not statistically significant. There was a significantly greater movement of the entire C7L3 segment, with the highest value of 3.99 deg. (SD 0.16) for the Hohmann brace and the lowest for TLSO at 1.71 deg. (SD 0.29). According to the questionnaire, the most comfortable brace was the Hohmann-type lace orthosis, with an average score of 4.50 (SD 0.15). The TLSO and Jewett braces were significantly less comfortable, with a score of 3.27 (SD 0.26) and 3.30 (SD 0.19), respectively.

## 4. Discussion

Spinal orthoses are popular comprehensive treatments for various spinal disorders. They present a leading therapy for moderate to severe idiopathic scoliosis during the developmental phase [6]. Spinal braces operate through two basic mechanisms of action. One approach is to increase the intra-abdominal pressure, thus reducing the net force applied to the spine and thus decreasing the stress exerted on the spine [7]. However, most orthoses provide a three-point fixation to maintain the spine in a favorable posture, which offloads some larger compressive forces [8]. The braces used in our study constitute both mechanisms: The Hohmann lace brace utilizes the former mechanism, the Jewett brace represents the latter type, and TLSO is a blend of both. Bracing of the spine is a choice method for the nonoperative treatment of thoracolumbar fractures [9], although its efficacy has often been challenged. A recent meta-analysis by Wallace et al. [10] failed to demonstrate any improvement in either clinical or radiographic outcomes. The study further claimed that using braces increases patient morbidity and incurs high costs without any clinical benefit. Bracing is also a predominant alternative for the treatment of vertebral osteoporotic compression fractures. However, this method has been under continuous scrutiny for years. The authors of a recent meta-analysis [11] showed that there is insufficient evidence to demonstrate the superiority of a rigid brace over a soft brace or no brace. Nevertheless, the most controversial yet widespread use of spinal bracing is in the treatment of non-specific low back pain, be it acute [12] or chronic [13]. Although it has been shown that orthoses improve posture and control in patients with non-specific low back pain [14], the evidence to support this application is scarce [15]. For years the braces were believed to cause trunk muscle weakness; however, this concept has not been proven by adequate evidence either [16,17].

Perhaps the major confounder in all studies concerned with the clinical efficacy of braces is the actual motion restriction that may or may not be related directly to the perceived rigidity of the orthosis. We designed our study to evaluate the orthoses through common everyday activities: standing, walking, and sitting. We also added the task of lifting an object from the floor as a test for extreme displacement. We chose this approach to reproduce the physiological loads acting on the orthoses while performing activities of everyday life. This approach differs from other studies concerned with the topic. The study by Lang et al. [18] investigated the maximum range of motion of thoracolumbar spine in 12 volunteers wearing three different braces with various levels of rigidity. The measurements were assessed using a 3D motion capture system. Their results supported the hypothesis that custom-made braces, being more rigid, provide better restriction of motion in all planes. We believe our approach resembles the conditions of the everyday life of patients when extreme displacements are rare. In a similar study, IMUs were used by Curfts et al. [5]; however, only a single sensor placed in the sacral region was used, and as it was not capable of sensor fusion, it provided data on an angular rate that was converted to attitude (inclination). The subjects in their study were asked to perform five routine tasks without wearing an orthosis and four different tasks while wearing one. In conclusion, the authors asserted that the most rigid braces provided the most effective motion restriction.

The assessment of orthoses with activities resembling daily living was made possible with the introduction of IMUs. They have become increasingly popular in the field of musculoskeletal research with their potential for novel studies. They provide a good alternative to laboratory-based systems, which provide data that are not applicable to real-life contexts [19]. IMUs are now commonly used for the clinical analysis of movement [20]. The MetaMotionR device used in the present study was validated for objective assessments of functional movement of the lumbar spine by Beange et al. [21]. The introduction of sensor fusion technology has significantly increased the prospects for future studies.

Despite various levels of perceived rigidity, the performance of the tested orthoses was comparable in the voluntary task of picking up an item from the floor. This is in contrast to more involuntary exercises such as walking and sitting. It has been proposed that spinal orthoses act by enhancing the awareness of one’s body posture via biofeedback [22]. It is possible that the orthoses worn by subjects performing more deliberate activities may trigger a contraction of lower back muscles, thus increasing the overall rigidity of the torso. The authors of a prospective study that evaluated the effectiveness of the dynamic corset versus the three-point brace [23] came up with an interesting hypothesis explaining the mechanism of action of less rigid spinal orthoses. According to them, the preservation of partial movement coupled with a dynamic behavior triggers biofeedback and activation of the paraspinal musculature. When the patient tries to bend forward, the device exerts a delicate pressure stimulating the dorsal muscles to contract. Broadly stating, softer braces do not provide factual support but act as a reminder to retain a correct posture. On the other hand, more sophisticated braces, such as TLSO, are cumbersome and impractical for everyday use except when indispensable. New intelligent materials [24,25] should provide better control of posture without compromising comfort.

## 5. Limitations of the Study

Although we perpetually strive for excellence, we also acknowledge our shortcomings. For instance, we have not performed a formal validation of sensors with the setup used in this study but rather took advantage of previous work executed by Zhou et al. [26], which found the sensors to be reliable and cost-effective. We took great care to firmly place the sensors. However, due to limitations in controlling the position of sensors under the brace, small changes in attitude were possible. Again, the study evaluated spine mobility in healthy subjects, which is vastly different from assessing real-life patients. On the other hand, this approach also reduces heterogenicity and allows focus solely on the mechanical properties of braces. Not least of all, we did not take into account the changes in abdominal pressure. Monitoring this value would have provided favorable support to the findings reported in the present study.

## 6. Conclusions

The measured rigidity of softer braces was significantly pronounced while performing voluntary tasks in comparison to rigidity observed in involuntary tasks. The available data do not allow exact explanations of this finding and further research is imperative.

## Figures and Tables

**Table 1:** The questionnaire on the comfort of each brace. Based on Curfts et al. [5].

Sl. No.	Criteria	Rating
1.	…is comfortable	1 2 3 4 5
2.	…causes no pain	1 2 3 4 5
3.	…does not pinch	1 2 3 4 5
4.	…can easily be worn under clothes	1 2 3 4 5
5.	…is easy and quick to use	1 2 3 4 5

## Data Availability

The data presented in this study are available on request from the corresponding author.
